# Immunoglobulin superfamily containing leucine-rich repeat (ISLR) negatively regulates osteogenic differentiation through the BMP-Smad signaling pathway

**DOI:** 10.1016/j.gendis.2023.101091

**Published:** 2023-09-09

**Authors:** Lei Xiong, Miaomiao Lan, Chang Liu, Lei Li, YingYing Yu, Tongtong Wang, Fan Liu, Kun Wang, Jin Liu, Qingyong Meng

**Affiliations:** State Key Laboratory of Agrobiotechnology, College of Biological Sciences, China Agricultural University, Beijing 100094, China

Osteoporosis (OP) is an aging-associated condition that significantly affects the quality of life in aging humans and has few treatment options. As an important marker, recombinant immunoglobulin superfamily containing leucine-rich repeat (ISLR) could be used as a gene therapy for OP. We herein used alkaline phosphatase (ALP) and alizarin red S (ARS) staining to evaluate osteogenic differentiation potential. Micro-computed tomography was used to determine bone formation and for image reconstruction of the trabecular distribution of the femur. ISLR knockout increased osteogenic differentiation and mineral deposition, consistent with the results in Ocn-cre:ISLR^flox/flox^ mice and pCMV6-Islr-GFP (ISLR-overexpression, ISLR-OE) mice. ISLR negatively regulates osteogenic differentiation through the bone morphogenetic protein 4 (BMP4)-Smad-ColIα1/Ocn axis, promoting proteasomal degradation of BMP4. ISLR interacted with BMP4 to regulate osteogenic differentiation. Moreover, interference of ISLR expression was potentially therapeutic for bone loss treatment. These results demonstrated a new mechanism of osteogenic differentiation that could be used for developing effective bone therapies to treat OP.

To study ISLR function, ISLR-deficient, ISLR^flox/flox^, and ISLR-OE transgenic mice were utilized, which were produced as previously described.^1^ The Jackson Laboratory (Bar Harbor, ME, USA) provided the Ocn-cre transgenic mice (Strain #:019509), which were then mated with ISLR^flox/flox^ mice in our institution. First, we discovered that levels of ISLR expression were high in bone, muscle, adipocytes, and the spleen in wild-type mice (Fig. S1B), indicating that ISLR plays a role in bone formation. Primary calvarial osteoblasts were isolated from the bone marrow, after which the long bone was broken down using collagenase I (Life Technologies, Thermo Fisher Scientific) and ethylene diamine tetraacetic acid. Our findings showed that ISLR knockout had no effect on osteogenic proliferation and that the number of Ki67^+^ primary calvarial osteoblasts showed no significant difference (Fig. 1A). ISLR knockout exhibited no difference in osteogenic proliferation, according to CCK8 tests as well. In six-month-old ISLR-deficient mice, trabecular bone volume/total volume (BV/TV), trabecular bone thickness (Tb.Th), and the number of trabecular bones (Tb.N) were all higher, and trabecular bone separation (Tb.Sp) was lower (Fig. S1C–G), as shown by ALP and ARS staining (Fig. 1B, C). ISLR knockout increases osteogenic differentiation potential. In Ocn-cre:ISLR^flox/flox^ mice, ISLR conditional knockout enhanced bone mass (Fig. 1D), whereas ISLR-OE mice had decreased bone mass (Fig. S2A–E), which is consistent with ISLR knockout mice having higher osteogenesis.

**Figure 1:**
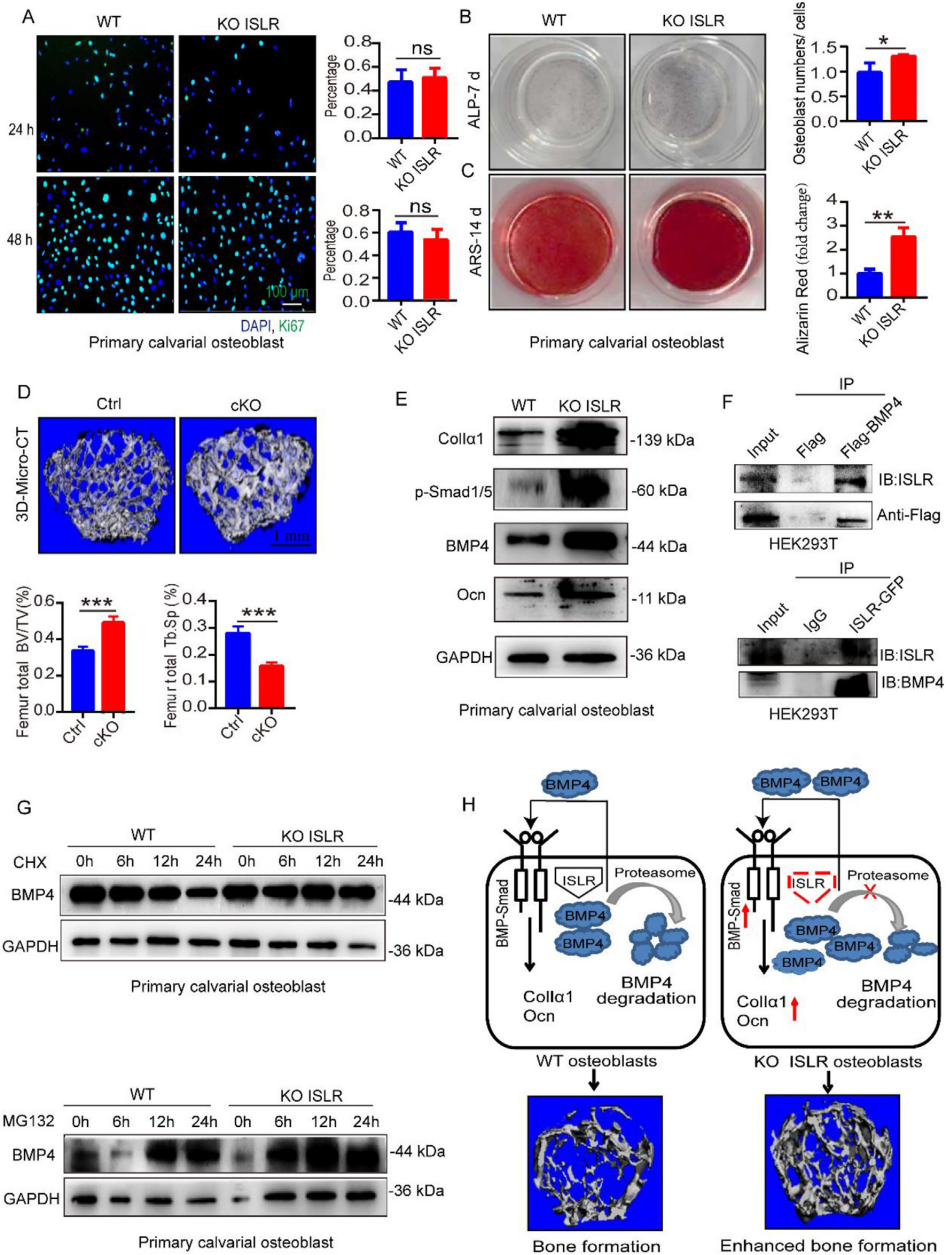
ISLR negatively regulates osteogenic differentiation through the BMP-Smad signaling pathway. **(A)** Ki67 immunostaining in primary calvarial osteoblasts cultured for 24 and 48 h, and the percentage of Ki67^+^ cells in wild-type and ISLR knockout primary calvarial osteoblasts at 24 and 48 h. **(B, C)** ALP and ARS staining in primary calvarial osteoblasts *in vitro* induced on days 7 and 14 of osteogenic differentiation. **(D)** 3D-Micro-CT analysis of the parameters (BV/TV, Tb.Sp) of the proximal femur in ISLR^flox/flox^ (Ctrl) and Ocn-cre:ISLR^flox/flox^ (cKO) mice. **(E)** ISLR knockout increased the protein expression of ColIα1 and Ocn in primary calvarial osteoblasts. **(F)** Interaction between ISLR and BMP4 was verified by immunoprecipitation in HEK293T cells. **(G)** Protein expression of BMP4 was detected in primary calvarial osteoblasts exposed to 50 μg/mL CTX and MG-132 for 0, 6, 12, and 24 h. **(H)** The mechanism underlying ISLR regulation of osteogenic differentiation through the BMP-Smad signaling pathway.

ISLR has been identified as a marker of osteogenic differentiation. The balance between osteoblastic and osteoclastic activities is regulated by the interplay between osteoblasts and osteoclasts.^2^ MSCs can multiply and develop into chondrocytes, osteoblasts, and osteocytes.^3^ ColIα1 and Ocn protein expression were increased by ISLR knockout, which also promoted osteogenic differentiation and mineral deposition. Skeletal diseases emphasize the significance of Runx2 activity during human osteogenesis, and Runx2 is also necessary for osteogenic differentiation. In this study, the expression of Runx2 protein was unaffected by ISLR knockout. The BMP/Smad signaling pathway is essential for bone formation. The results demonstrated that ISLR regulates osteogenic differentiation through the BMP4-Smad-ColIα1/Ocn axis (Fig. 1E; Fig. S2F, G).

Alternatively, p-Smad1/5 expression was increased by rhBMP4 more than rhBMP7, and rhBMP2 and rhBMP4 had the greatest ability to promote bone formation.^4^ Furthermore, two GEO profiles revealed that BMP4 treatment of C3H10T1/2 cells or embryonic AGM VE-cadherin^+^ cells increased ISLR expression. Therefore, we focused on the BMP4 protein, and we discovered that ISLR interacted with BMP4 to promote its proteasomal degradation. Ocn and ColIα1 protein expression was elevated by ISLR knockout to support osteogenesis. Since OP induces a persistent reduction in bone formation, we hypothesized that blocking ISLR expression could prevent bone loss and serve as a therapeutic strategy for OP treatment. Besides, Xu et al^5^ also reported that in the stromal epithelium, the ISLR-YAP signaling axis is essential for modulating epithelial cell growth during intestinal regeneration. As MSCs differentiate into different cells and organs, ISLR has been discovered as a potential marker for these cells. In osteogenic differentiation and bone formation, a novel ISLR-regulated mechanism was discovered that might be targeted to provide secure and efficient bone therapeutics.

The mechanism underlying ISLR regulation of osteogenic differentiation through the BMP-Smad signaling pathway is shown in Figure 1H. Mechanistically, the BMP-Smad signaling pathway is involved in osteogenic differentiation, and ISLR interacted with BMP4 to induce proteasome-dependent degradation during normal bone remodeling (Fig. 1F, G). The interaction between ISLR and BMP4 during typical bone remodeling is restricted by ISLR knockout. Through the BMP-Smad signaling pathway, ISLR knockout promoted osteogenic differentiation, reduced the proteasome-dependent degradation of BMP4, and increased the expression of ColIα1 and Ocn. Additionally, ISLR knockout enhanced osteogenic differentiation, thereby increasing bone mass. Thus, ISLR negatively regulates osteogenic differentiation through the BMP4-Smad-ColIα1/Ocn axis.

In conclusion, our data indicated that ISLR knockout mice displayed increased osteogenesis. ISLR knockout had no significant effect on osteogenic proliferation. ISLR knockout increased osteogenic differentiation and mineral deposition. ISLR negatively regulates osteogenic differentiation through the BMP4-Smad-ColIα1/Ocn axis. ISLR interacted with BMP4 to regulate osteogenic differentiation, promoting the proteasomal degradation of BMP4. Specific deletion of ISLR in osteoblasts increased bone mass in Ocn-cre:ISLR^flox/flox^ mice. However, bone mass was decreased in ISLR-OE mice. Thus, ISLR is a novel regulator of osteogenic differentiation and bone development.

## Ethics declaration

The animal study protocols were approved by the Institutional Animal Care and Use Committee of China Agricultural University (approval number: AW12906102-3-1).

## Author contributions

L.X. and Q.M. conceived the experiments, analyzed the data, and wrote the manuscript. M.L., C.L., L.L., Y.Y., T.W., and F.L. provided reagents and generated animals. K.W. and J.L. contributed to the review and editing of the manuscript and provided expertise and feedback. Q.M. secured funding.

## Conflict of interests

The authors declare no conflict of interests.

## Funding

This study was supported by the 10.13039/501100001809National Natural Science Foundation of China (No. 31790412, 31970712), the 10.13039/501100012166National Key Research and Development Program of China (No. 2021YFF1000603), and earmarked fund for CARS36, the Plan 111 (B12008).
