# Supplementary material for: Immunoglobulin superfamily containing leucine-rich repeat (ISLR) negatively regulates osteogenic differentiation through the BMP-Smad signaling pathway
Source: Genes Dis. 2023 Sep 9;11(4):101091. doi: 10.1016/j.gendis.2023.101091 (PMC10955205; doi:10.1016/j.gendis.2023.101091)
Supplement: Multimedia component 1 [file mmc1.docx]

**Supplementary Data**

**Immunoglobulin superfamily containing leucine-rich repeat (ISLR) negatively regulates osteogenic differentiation through the BMP-Smad signaling pathway**

Lei Xiong^1^, MiaoMiao Lan^1^, Chang Liu^1^, Lei Li^1^, YingYing Yu^1^, TongTong Wang^1^, Fan Liu^1^, Kun Wang^1^, Jin Liu^1^, Qingyong Meng^1^*


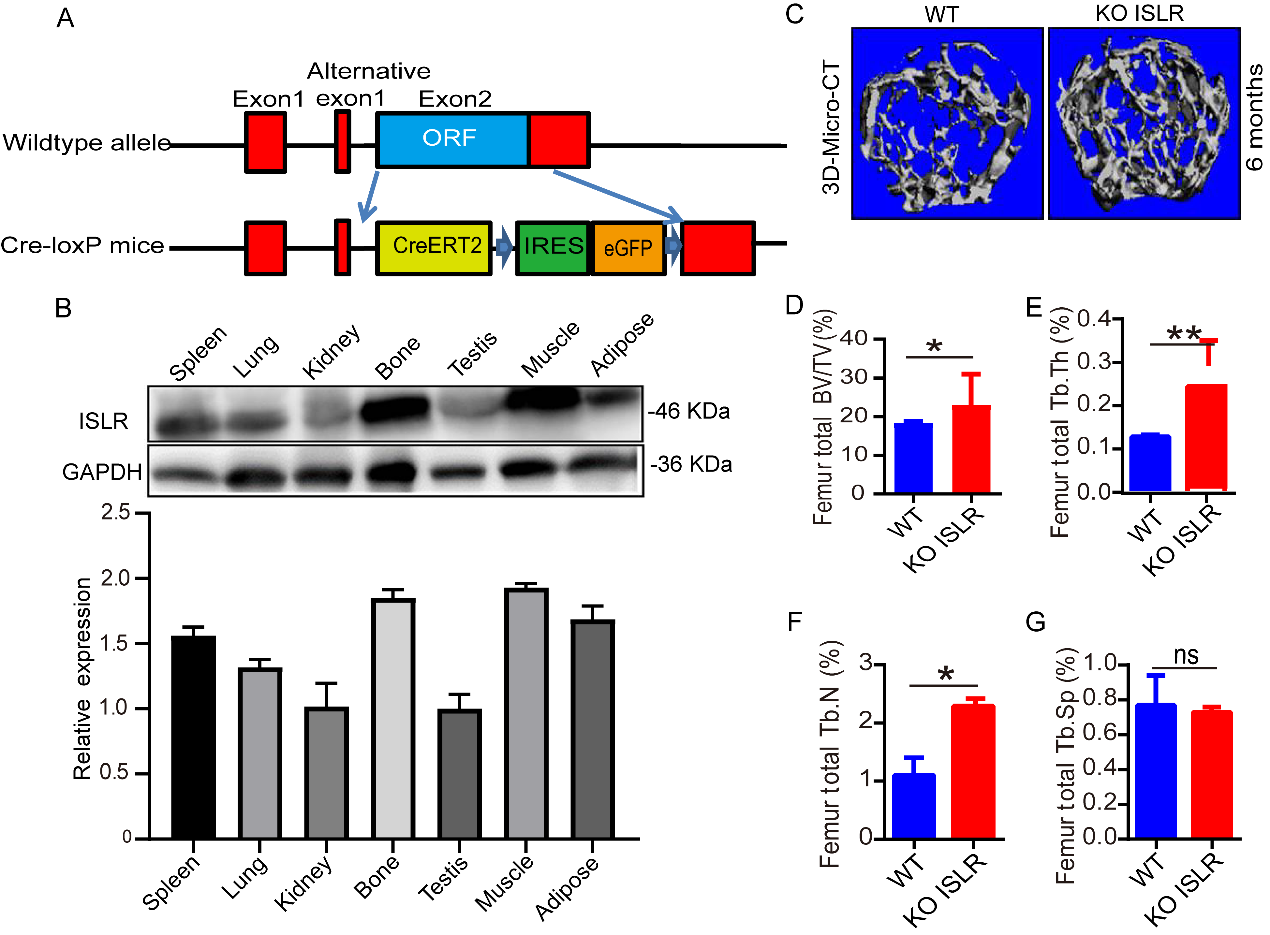


**Figure S1. ISLR-knockout mice displayed increased osteogenesis.** **(A)** Pattern of ISLR expression in ISLR knockout mice. **(B)** Expression of ISLR in the spleen, lungs, kidneys, bones, testes, muscles, and adipose tissues. **(C)** Micro-CT analysis. **(D–G)** Parameters of the proximal femur (BV/TV, Tb.Th, Tb.N, and Tb.Sp) in wild-type and ISLR knockout mice.


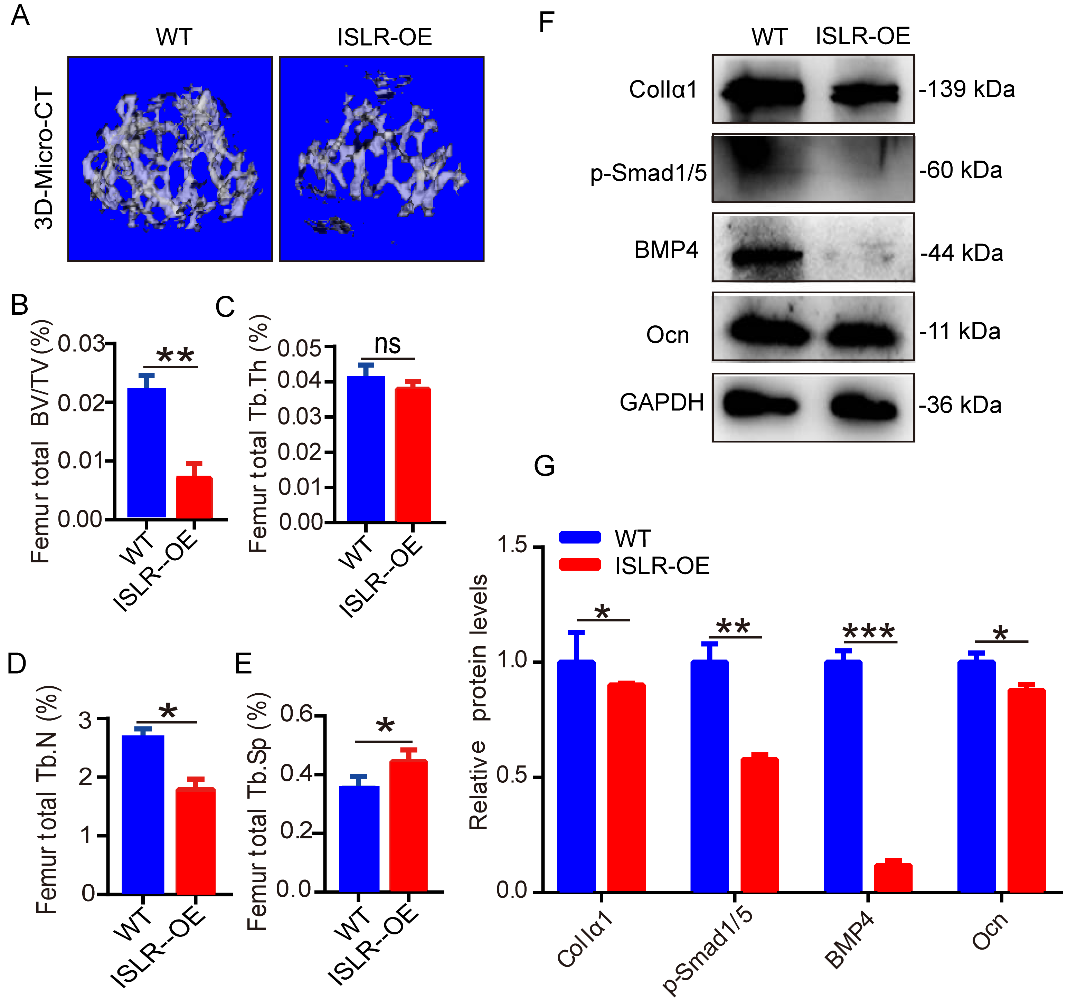


**Figure S2. Bone mass was decreased in ISLR-OE mice. (A)** Micro-CT analysis. **(B**–**E)** Parameters of the proximal femur in wild-type and ISLR-OE mice were detected. **(F**, **G**) The effect of ISLR-OE on the BMP-Smad signaling pathway, and the protein expression of ColIα1 and Ocn was detected.

**Supplemental Materials and methods**

**Micro-computed tomography (CT) analysis**

The femur was analyzed in mice using the NEMO Micro CT system [NMC-100, PINGSENG Healthcare (KunShan), Inc., Jiangsu, China] at a resolution of 10 μm. The proximal femoral bone parameters were analyzed using the instrument’s analysis software, including trabecular bone volume/total volume (BV/TV), trabecular bone thickness (Tb.Th), the number of trabecular bones (Tb.N), and trabecular bone separation (Tb.Sp). The scanning tube voltage was set to 60 kV, and the tube current was set to 120 μA. Each selected slice was segmented for three-dimensional reconstruction to calculate bone formation parameters. The volume of interest was defined as the cylindrical area that covered the initial bone.

**Western blot analysis**

We used RIPA buffer containing protease and phosphatase inhibitors to extract cell and tissue proteins. Proteins were separated by 10% sodium dodecyl sulfate-polyacrylamide gel electrophoresis and then transferred to a polyvinylidene difluoride membrane at 4°C (Life Technologies) using a wet transfer apparatus. A 5% fat-free milk was used to block the membranes for 1 h, followed by incubation with primary antibodies against BMP4 (1:1000, Abcam, Cambridge, UK) and p-Smad1/5 (1:1000, Cell Signaling Technology, Boston, MA, USA) at 4°C overnight and then with the secondary antibody. Immunoreactive bands were visualized, and densitometric analysis was performed using ImageJ software. GAPDH was used as a loading control.
